# Ventricular Septal Rupture Management in Patients With Acute Myocardial Infarction: A Review

**DOI:** 10.7759/cureus.40390

**Published:** 2023-06-13

**Authors:** Apoorva Tripathi, Himanshi Bisht, Akshat Arya, Ashwati Konat, Divya Patel, Jay Patel, Dhruvin Godhani, Kamalika Mozumder, Dhyey Parikh, Pragya Jain, Kamal Sharma

**Affiliations:** 1 Medicine, Byramjee Jeejeebhoy Medical College, Ahmedabad, IND; 2 Internal Medicine, Byramjee Jeejeebhoy Medical College, Ahmedabad, IND; 3 Department of Zoology, Biomedical Technology and Human Genetics, Gujarat University, Ahmedabad, IND; 4 Trauma and Orthopaedics, Gujarat Medical Education and Research Society Medical College, Gandhinagar, IND; 5 Internal Medicine, Gujarat Medical Education and Research Society Medical College, Gandhinagar, IND; 6 Internal Medicine, Smt Nathiba Hargovandas Lakhmichand Municipal Medical College, Ahmedabad, IND; 7 Cardiology, Dr. Kamal Sharma Cardiology Clinic, Ahmedabad, IND

**Keywords:** intra-aortic balloon pump, mechanical circulatory supports, percutaneous closure device, myocardial infarction, ventricular septal rupture

## Abstract

Untreated myocardial infarction (MI) can potentially lead to many fatal complications which require immediate management. One of them is ventricular septal rupture (VSR) which necessitates the hemodynamic stabilization and closure of the septal rupture. Conventional treatment strategy involves surgical repair; however, percutaneous transcatheter repair using an occluder device is a promising upcoming approach. We conducted a detailed review of various published articles and examined the trends in incidence, risk factors, and pathophysiology of MI leading to VSR followed by an in-depth analysis of the various management strategies for the same. In the current clinical scenario, thrombolysis is an imperative management strategy that has been shown to decrease the occurrence of VSR by manifolds, more specifically in patients having ST-elevated MI. Delayed surgical closure remains the main treatment for post-infarction VSR. Other newer modalities, such as percutaneous closure devices and mechanical circulatory supports, are attractive alternative or complementary strategies to treat such patients, both postoperatively and perioperatively. However, earlier surgical repair in VSR increases the risk of mortality, and the optimal timing for VSR closure remains controversial. Despite surgical closure of VSR being the traditional treatment, it presents a considerably high operative risk. Although newer interventions such as percutaneous closure devices and mechanical circulatory supports provide impressive outcomes, their efficacy in high-risk patients remains inconclusive.

## Introduction and background

Ventricular septal rupture (VSR) is a surgical emergency complicating acute myocardial infarction (AMI) calling for a closure of the septal defect along with coronary artery bypass grafting in symptomatic patients. Surgery is also indicated in asymptomatic patients depending on the size of the defect [1]. VSR in current times is quite rare. It was earlier linked to the traditional methods of reperfusion and had a high prevalence of 2%. With the initiation of newer methods of thrombolysis and reperfusion involving percutaneous coronary intervention (PCI), the incidence has decreased dramatically to 0.31% [2,3]. Although the incidence has reduced, the mortality rate still remains quite high at about 41%-80% without any evident decline in the past few decades making post-MI VSR a cause for concern [4].

Of the 13,767 patients in the study reported by Elbadawi et al. who experienced mechanical complications, 10,344 (75%) patients went on to develop a VSR, while the remaining patients went on to develop papillary muscle rupture and free-wall rupture [5]. As an effective treatment modality over the past few years, a patient with post-infarct VSR is managed by a “Heart Team” of cardiothoracic surgeons and interventional cardiologists who perform surgery via a trans-infarct approach [1]. Mechanical circulatory supports (MCSs) and percutaneous closure devices may provide alternate or complementary approaches to treating these patients perioperatively [6,7].

In all of its stages, from preoperative to postoperative, post-infarction VSR continues to be a serious complication demanding care. Early loss of life remains a challenge despite significant improvements in AMI treatment and surgical procedures for VSR repair. According to a recent analysis, the treatment approach being adopted currently has not resulted in significant improvements in outcomes over time [8]. Moreover, there is a need to evaluate and analyze in depth the various management strategies of VSR and their ambiguous aspects to curtail the mortality associated with VSR. Hereby, we review the various studies discussing the management of VSR.

## Review

Ventricular septal rupture

Given the fatality of VSR as a complication in post-MI patients, it is imperative to discuss the various aspects of VSR, such as incidence, pathophysiology, morbidity, mortality, and management. VSR is known to be caused by an infarct of the interventricular septum which separates the two ventricles of the heart. VSR involves coagulation necrosis that occurs due to the denaturation of proteins caused by a reduction in oxygen supply to the myocardial tissue [1]. Recent studies suggest that VSR develops between three and five days after an episode of AMI, generally developing within three days [2]. Regarding pathophysiology, the primary mechanism behind VSR is shearing forces between the healthy myocardium and the infarcted area. These physical stressors might further cause ventricular aneurysms, papillary muscle rupture, and free wall rupture.

The part of the interventricular septum that gets ruptured and the resulting type of VSR depends on which artery is the site of full-thickness MI. Table 1 shows the arteries involved and the corresponding part of the interventricular septum that gets affected along with the type of VSR [9].

**Table 1:** Affected artery corresponding to the type of VSR. VSR: ventricular septal rupture

Coronary artery affected	Interventricular septum part affected	Type of VSR
Left anterior descending coronary artery	Anterior portion	Apical VSR
Dominant right coronary artery	Inferior portion	Basal VSR
Dominant left circumflex artery	posterior portion	Unknown

VSR can seldom be misdiagnosed as acute mitral regurgitation, tricuspid regurgitation, and acute flash pulmonary edema. VSR can be associated with free wall rupture, ventricular aneurysms, and cardiogenic shock and can often lead to death. The high mortality associated with VSR makes it crucial to investigate and study in depth the impact factors associated with it. One such impact factor is the incidence and epidemiology of VSR. As cited by the Global Registry for Acute Coronary Events (GRACE) study, AMI patients who were treated with percutaneous intervention in the first place tend to be at a lesser risk for VSR (0.7%) compared to patients treated with thrombolytic therapy (1.1%) [10]. An important fact to be noted regarding the incidence of VSR is that whether the infarct is present in the anterior wall or the inferolateral wall; however, either of them will have similar consequences leading to VSR. Further, ST-segment elevation MI is more associated with VSR incidence.

Almost all VSR patients present with recurrent chest pain and discomfort. On auscultation, a loud systolic murmur is heard. Owing to an increased gush of blood, a loud second heart sound can be heard. Patients may also present with flash pulmonary edema and in severe cases may present with cardiogenic shock. The risk factors for VSR include female gender, chronic kidney disease, old age, high GRACE score, and ST-elevation MI. If done timely and efficiently, evaluation and diagnosis can lead to a better prognosis for the patients. A chest X-ray may demonstrate an enlarged left ventricle and pulmonary edema [11]. However, transthoracic echocardiography (TTE) is the gold standard diagnostic method; thus, for the final confirmation of VSR, two-dimensional TTE needs to be done which is useful in finding out the precise anatomical size and site of rupture in the ventricular wall [12]. Color Doppler echocardiography is of help in determining the blood flow across the ventricular walls [12]. In case of an inadequate view of the myocardium due to TTE, the transesophageal method can also be implemented for an echocardiogram, as seen in patients on mechanical ventilators. Moreover, to differentiate VSR from mitral regurgitation, cardiac catheterization can be considered as VSR patients demonstrate a step-up of oxygen between the right atrium and the pulmonary artery [13]. Winking coronary sign of VSR (transient systolic occlusion of infarct related to the artery overlying the VSR) has also been described on coronary angiography [14].

VSR is a fatal condition with extremely high mortality. Patients with cardiogenic shock experience the worst prognosis [15]. Despite the unfavorable prognosis, early repair of VSR is tricky as the sutures made do not stay in place in soft and friable tissue. A delay becomes necessary as fibrosis makes it conducive for the suturing to occur. Advanced age, multiple organ failures, and posterior VSR are some of the factors that result in worse prognoses. A patient is more likely to die within 30 days if they present with any of the conditions such as renal failure, shock, and significant coronary disease. However, the prognosis is comparatively less fatal in the case of an anterior infarct and smaller rupture size [16].

Treatment

Management of VSR can be done using two modalities: apart from the medical management, the first option is the surgical approach, and the second is the interventional device closure.

The primary goal of medical treatment for post-infarct VSR should be hemodynamic stabilization and, where allowed, afterload reduction using vasodilators [17]. Vasodilator therapy using nitroglycerine or nitroprusside must be initiated as soon as possible if systolic blood pressure is greater than 90 mmHg [18]. Inotropes might be needed to support a sufficient cardiac output to stabilize the patient’s state and set the stage for additional repair [18]. Intra-aortic balloon pump (IABP) is utilized, especially in the event of unsuccessful pharmaceutical therapy [19,20]. Mortality is independently linked with preoperative percutaneous revascularization, including thrombolysis and PCI, which may be connected to the greater hemorrhagic risk following such procedures, although further research is needed to confirm this [21].

Although considered the backbone of therapy, surgery is occasionally delayed with the use of mechanical support but the majority of patients undergo urgent surgery owing to the frequent complications that include poor clinical condition (particularly shock), pulmonary overcirculation, and multiorgan failure to obtain a more effective repair [17]. Using a route through the right ventricular outflow system, Cooley and colleagues described the first patient who was surgically treated [22]. Later, other techniques were developed that offered more promising results. A technique described by Willard M. Daggett, Jr involved infarctectomy and direct closure of the left VSR, with or without a patch which became quite popular until Tirone David introduced a new technique that excluded the need for an infarctectomy [23]. Studies have shown that David infarct exclusion repair is better in comparison to the Daggett repair [23]. This method does not provide sufficient access to the entire septum, and a few years later, the majority of surgeons began using an incision through the infarcted left ventricle [24]. Regardless of the hemodynamic condition, the current American College of Cardiology recommendations advocate for early surgical repair [25]. However, at times, postponement of surgical repair is done in favor of more medical stabilization and circulatory customization; this can occasionally take weeks for patients who are not readily accepted. A delay has the effect of allowing cardiac restructuring and tissue repair, especially at the edges of the defect, but patients who declare themselves to be clinical survivors are able to get definitive therapy while those who are moribund are not [26-29]. Regardless, mortality rates and repeated shunting rates remain high after surgical repair, which may be related to patch leaks caused by sutures coming loose from infarcted tissue [30].

The first report on an interventional strategy for post-infarct ventricular septal defect (VSD) closure was published in 1988 by Lock and colleagues [31]. Transcatheter therapy with device occlusion has been investigated as a different management option in several situations, including (1) as an acute therapy within three to five days after VSR is noted, (2) as a subacute therapy after allowing tissue remodeling and fibrosis, or (3) as a salvage therapy after patch repair/infarct exclusion where a residual substantial shunt exists [17]. The surgery for VSR can be quite challenging. The timing of the closure and whether it is performed as a primary VSR closure versus post-surgical repair are crucial factors to take into account when assessing device series for post-infarct VSR closure [17]. Only sporadic case reports and a few case series are used as available evidence for transcatheter closure of post-infarct VSR [17].

For big defects in unstable patients, surgery is preferable to device therapy as the first-line treatment for post-infarct VSR. Nevertheless, many patients are not good surgical candidates due to age, comorbidities, multiorgan failure, hemodynamic instability, inefficiency, or patient preference [17]. Device treatment appears to be a good option for sealing post-surgical VSR patch leakage. Owing to this, a temporary hybrid strategy with early surgical repair and device closure of a patch leak, should it occur, seems reasonable. Offering device therapy as a last resort to a subset of patients who are considered inoperable is still up for debate [17].

Although several devices can be used to close VSR, the AMPLATZER™ PIVSD device, which has a maximum size of 24 mm, has garnered maximum usage. This device is flawed because it frequently does not conform to the irregular and jagged edges of a VSR, requires a somewhat large sheath to deliver, and is quite stiff and cumbersome [17].

A varied number of complications have been reported related to this procedure, including ventricular fibrillations/tachycardias, transitory third-degree atrioventricular blocks, third-degree atrioventricular blocks after crossing the VSD with the delivery sheath with consecutive death before pacing implementation, electromechanical dissociation with subsequent death, atrial flutter terminated by cardioversion, several other arrhythmias of varying degrees, and unspecified bradycardias linked to the implantation of the VSD occluder [32-38].

To assess the efficacy of percutaneous closure of post-infarction VSD, Schlotter et al. [38] performed a systematic literature review. They discovered 13 series with at least five instances per report with a total of 273 patients [39]. Cardiogenic shock was evident in 48% of patients, interventions were performed within two weeks of rupture in 42% of instances, and 89% of device implantations were effective. Overall, 32% of patients who were effectively treated died in hospitals [39]. Recent studies have shown that interventional VSD closure is an effective procedure [33-38,40-44]. Regarding developing standards for proper suitability, currently, there is no unanimous agreement [39].

Percutaneous device closure of VSD offers the benefit of early shunt lowering to minimize hemodynamic deterioration and may be a worthwhile alternative to surgical correction given that these procedures can be performed with a high degree of technical success [39]. Post-infarction VSD has a terrible prognosis if it is not treated. The majority of patients receiving primary device therapy for acute conditions are reported to have increased mortality rates, mostly as inpatients due to multiorgan failure rather than from problems connected to the devices; nevertheless, those who do survive tend to fare better in the medium to long term. Hence, percutaneous device closure of post-MI VSD can be a good alternative to surgical repair. Due to the lack of randomized data, a multidisciplinary team must evaluate the clinical choice of the type of treatment (surgical versus interventional approach) based on the complexity of the defect [39]. This is a promising procedure that offers a high degree of precision and success but remains an unexplored and unconquered territory that needs further exploration. Adverse events are still very poorly understood, and the decision to proceed with this management plan needs to be made keeping in mind the patient profile as well as the experience of the operating personnel. Of late, there have been case reports of using ASD closure devices that entails a possibility of lower risk of hemolysis due to its better hemodynamic profile compared to VSD closure devices [45].

## Conclusions

One of the most effective ways to treat VSR for better survival results continues to be surgery. Nonetheless, the timing of surgery and advanced stages of shock at presentation also significantly impact the prognosis for VSR patients. Early mortality has been shown to be quite high despite numerous improvements in the treatment of AMI and in the surgical methods of VSR repair, with no signs of improvements during the previous 20 years. Although transcatheter closure of VSR can be performed with great technical success and very few procedural complications, it is linked to very high in-hospital death rates in the acute context. Patients who are not candidates for surgery but who typically have hemodynamically acceptable abnormalities and survive a period of watchful waiting may also be given consideration for transcatheter closure. Patients who are treated subacutely and make it to hospital discharge appear to have good long-term outcomes. Despite the fact that there have been considerable improvements in the treatment of VSR, it is clear that more research is needed to fill in the knowledge gaps that still exist and to identify creative approaches that can improve patient outcomes. We can only expect to improve and optimize the management strategies for this complicated ailment through ongoing research and collaboration, ultimately giving patients in need a better future.
